# Feelings of worthlessness links depressive symptoms and parental stress: A network analysis during the COVID-19 pandemic

**DOI:** 10.1192/j.eurpsy.2021.2223

**Published:** 2021-07-27

**Authors:** Nora Skjerdingstad, Miriam Sinkerud Johnson, Sverre Urnes Johnson, Asle Hoffart, Omid V. Ebrahimi

**Affiliations:** 1 Department of Psychology, University of Oslo, Oslo, Norway; 2 Department of Behavioural Science, Oslo Metropolitan University, Oslo, Norway; 3 Research Institute, Modum Bad Psychiatric Hospital, Vikersund, Norway

**Keywords:** COVID-19, depression, mechanisms, network analysis, parental stress

## Abstract

**Background:**

The prevalent co-occurrence between parental stress and depression has been established prior to and during the COVID-19 pandemic outbreak. However, no studies to date have identified the connections through which these symptom domains interact with each other to emerge into a complex and detrimental mental health state, along with the plausible mechanistic variables that may play key roles in maintaining parental stress and depression. The aim of this research is to uncover these interactions in a period where parents experience heightened demands and stress because of the strict social distancing protocols.

**Methods:**

Network analysis is utilized to examine parental stress and depressive symptoms during the COVID-19 pandemic in a large cross-sectional study (*N* = 2,868) of parents. Two graphical Gaussian graphical network models were estimated, one in which only parental stress and depression symptoms were included, and another in which several mechanistic variables were added.

**Results:**

Expected influence and bridge expected influence revealed that feeling worthless was the most influential node in the symptoms network and bridged the two psychological states. Among the mechanistic variables, worry and rumination was specifically relevant in the depressive cluster of symptoms, and self-criticism was connected to both constructs.

**Conclusion:**

The study displays that the co-occurrence of parental stress and depression has specific pathways, was manifested through feelings of worthlessness, and has specific patterns of connection to important mechanisms of psychopathology. The results are of utility when aiming to avoid the constellation of co-occurring parental stress and depressive symptoms during the pandemic.

## Introduction

Being quarantined and isolated at home has been our new everyday life, as the novel COVID-19 evolves globally, and governments implement social distancing measures to reduce the spread of the virus. As infection and death rates increased and citizens sheltered at home during the initial phases of the COVID-19 pandemic, the general population have experienced elevated psychological distress (e.g., [1–4]). Research on the parental population have outlined high prevalence of clinically significant levels of depression compared to prepandemic levels [5,6] in addition to heightened parental stress [6–10].

Parental stress, which refers to a mismatch between parenting demands and the available resources to deal with these demands [11], may have adverse psychological implications for the individual parent, their partner, and their children. Associations between parental stress and depressive symptoms have been demonstrated (e.g., [12–15]).

Depressive symptoms and parental stress are serious mental health states that may reduce parents’ abilities to support and connect to their children’s mental development, putting the offspring at increased risk of experiencing a range of mental disorders (e.g., [16]). Previous findings have discovered that stress related to being a parent is associated with depressive symptoms in mothers and fathers during nonpandemic periods [17–20] and during the ongoing pandemic (e.g., [6,7]). Studies, mainly on mothers with depression, have indicated that parental stress is elevated in mothers with depression compared to those without [12]. A longitudinal study on the predictors of parental stress revealed that earlier parent-related depression predicted levels of parental stress [14]. This pattern in the literature leaves a trail of global associations between the constructs, but yet unmapped relationships between specific components of parental stress and how they interact with symptoms of depression, specifically relevant for investigations through the network approach.

The network approach to psychopathology represents a unique view of psychological symptoms and how they interact in complex systems [21]. The perspective embodies the possibility to explore how depression and parental stress are intertwined in a network of components (nodes), shedding light on potential bridging and central nodes in this co-occurring structure [22–24]. To this date, no study has investigated the network structure of parental stress and depressive symptoms, neither in pandemic nor in nonpandemic settings. The current research will thus investigate this research question in relation to the initial stages of the pandemic; a time of great uncertainty, and strict implementation of strategies to impede the infection rates, including school, kindergarten, university, and workplace lockdowns, closure of public spaces, and a general encouragement to shelter at home. Such situation represents an unusual setting in which parents experience a range of new external stressors. Such external stressors, also referred to as events in the external field, have the possibility to disrupt the stability of a system, leading establishment of new connections and the strengthening of previous connections in ways which may emerge into novel complex mental health symptoms co-occurrences [22].

Several mechanistic variables, which are theorized to emerge forth parental stress and depressive states, were included in the analysis to explore preliminary associations of such mechanisms with the established network structure. Mechanistic variables refer to strategies that participants use to cope with difficult emotions and/or symptoms [25]. Different therapeutic approaches build their interventions around such mechanistic variables with the rationale that modification of these processes may ameliorate detrimental mental health states. For this study, the following mechanisms were explored: (a) adjusting behavior with alcohol or other substances, (b) excessive worry and rumination (WR) to cope with negative situations and feelings, (c) reduced ability to control behavior, and (d) self-criticism (SC).

The mechanisms are theorized to be significant maintaining mechanisms that may interact and contribute to a reinforcing parental stress and depressive system. Initial findings from the general population highlight excessive WR to be involved in depressive symptomatology [26,27]. Rumination, constituting of the time spent on repetitive and passive “dwelling” on problems, is found to be maintaining mechanism in many forms of psychopathology [28].

Self-critical thinking concerns negative beliefs about oneself and is involved in depressive states (e.g., [29,30]), and in parental stress and depression particularly (e.g., [31–33]). The increased demands that parents experience during the pandemic have heightened the pressure to fulfill their role as parents, electing more self-doubt and SC, especially when the obstacles are met in a vacuum with limited social support and help. Individuals experience the need to fulfill roles as teachers, employees, and parents simultaneously, possibly eliciting a feeling of not being able to handle the increasing number of tasks and responsibilities.

In the light of recent worries regarding elevated alcohol use among parents during the pandemic [34,35], this paper investigates using alcohol to cope with one’s negative feelings and emotions during the pandemic, a strategy that previously found to be elevated in parents during in the present pandemic context [35]. Investigating the associated symptoms of such coping strategy is thus of immediate importance, given the potential influence it may have on parenting and adverse mental health constellation. In addition, in a time where families spend most of their time at home, parents’ emotion regulation abilities are crucial to preserve a healthy family dynamic and reduce conflicts. Consequently, impulse control difficulties, specifically the reduced ability to control behavior, were examined.

Taken together, no studies have to date identified the specific connections between parental stress and depressive symptomatology and their interaction with mechanistic variables that could play key roles in the maintenance of parental stress and depression. Thus, the aim of this study is to investigate how these theorized variables interact with parental stress and depressive symptoms during the pandemic, which is of immediate utility due to aggravation of these problems during the COVID-19 pandemic. Given the high occurrence of the problematic states prior to the pandemic, this study will nonetheless add to the general understanding of how the constellation of the interwoven mental constructs comes forth.

## Methods

### Sample and data collection

This research is part of the Norwegian COVID-19, Mental Health and Adherence Project [1]. Ethical approval of the study was granted by the Regional Committee for Medical and Health Research Ethics (reference number: 125510) and the Norwegian Centre for Research Data (reference number: 802810), where the study protocol and analysis plan were approved prior to data collection.

The study utilized a cross-sectional design in obtaining the mental health symptoms in the general parental population during the strict government-initiated social distancing protocols in the beginning of the pandemic outbreak in Norway. The protocols were implemented on March 12, 2020, and the 7 days of data collection lasted from March 31, 2020, to April 7, 2020. Three weeks prior to and during the data collection period, the social distancing protocols were held constant. Neither new information or modification of protocols was given by the government during this period, keeping the effect of changing protocols and expectation effects constant. The implemented protocols included lockdowns of kindergarten, schools, and universities, workplaces, and other public spaces, limited social contact and prohibitions of social gatherings and public events, and travel restrictions. Eligible participants in this study included adult parents (>18 years) living with one or more child(ren) at home, who were residing in Norway, and who provided informed consent to partake in the study.

The online survey was disseminated through broadcasting on national, regional, and local information platforms (i.e., television, radio, and newspapers). In addition, the survey was distributed to a random selection of Norwegian adults through a Facebook Business algorithm, thus ensuring that a representative sample of parents was invited to partake in the study. Further details about the data collection can be found elsewhere [6]. There were no data missing as participants were prompted to complete skipped items, if any.

### Measures

Demographic data included age, sex, number of children, work situation, and civil status. All measures of parental stress and depressive symptoms were included through a consensus procedure following discussions by clinical experts with the aim of avoiding topological overlap [36]. All single items included in the network analyses (19 items) are attached in the Supplementary Materials.

Depressive symptoms were measured with the Parent Health Questionnaire-9 (PHQ-9) [37]. The PHQ-9 consists of nine items aimed to cover the DSM-IV criteria for major depression, and participants were asked to rate the statements on a four-point Likert scale ranging from “not at all” (0) to “almost every day” (3) based on the respondents’ evaluation of the last 2 weeks.

Parental stress was measured with three items from the Danish Parental Stress Scale [38]. The questionnaire is developed as a short measure aimed to capture the perceived stress parents experience in their parental role. With the questions concerning the last 2 weeks, parents were asked to rate the statements on a five-point Likert scale ranging from “strongly disagree” (1) to “strongly agree” (5). In addition, measures of parents’ frustration and anger toward their children, parental guilt (PG), and feelings of inadequacy as a parent were included in the network evaluated on the same timespan (last 2 weeks) and Likert scale.

Measures of WR, SC, difficulty to control behavior, and use of alcohol or pills to deal with negative emotions were included to obtain relevant maintenance factors associated with depressive symptoms and parental stress. WR were measured using a single item from the Cognitive-Attentional Syndrome Questionnaire-1 (CAS-1) [28]. In addition, an item measuring the use of alcohol, pills, or drugs to cope with negative emotions was selected from the CAS-1. Here, respondents were asked to rate the statements about the last weeks on a nine-point Likert scale ranging from “nothing” (0) to “all the time” (8). SC and difficulty to control behavior were also included as single items in the network. These items were formulated as general functioning behaviors and scored on a four-point Likert scale ranging from “not at all” (0) to “almost every day” (3) for SC, and a five-point Likert scale ranging from “almost never” (1) to “almost always” (5) for behavior control.

### Statistical analyses

Statistical analyses were conducted in R (version 4.0.2; R core Team, 2019) [39]. The complete R-code can be found in the Supplementary Materials.

Nonparanormal transformations were applied to the dataset to deal with skewed data (see Supplementary Table S1) using the R package *huge* [40]. In order to ensure that none of the variables included in the network overlapped conceptually, a data-driven method for identifying potentially redundant nodes was added on top of the aforementioned consensus procedure, as reported in, for example, Blanchard et al. [41]. Here, the correlation matrix was checked and confirmed to be positive definite, controlling for linear combinations among the variables (see Supplementary Figure S1). Next, the *goldbricker* function in the R package *networktools* [24] was used to identify particularly redundant variables. A method previously offered by Hittner et al. [42] was applied for comparing dependent correlations, and the results returned a recommendation to not remove any variables from the analysis (no redundant variables identified), thus providing further support for the validity of the theoretical selections and consensus procedure conducted.

Given the large number of participants (*N* = 2,868) and in accordance with recent recommendations [43] addressing which estimation method to use for the research question of interest (bridging two constructs), unregularized graphical Gaussian model were used to estimate the network structures. Here, nodes represent depressive and parental stress symptoms, and the edges between them represent partial correlation between variables when all other variables are held constant. Two network models were estimated: one including only parental stress and depressive variables to reveal the mental health state network; and a second including the mentioned four mechanistic variables. Following recent recommendations [44], the current network estimation is based on the *ggmModSelect* method in the R package *qgraph* [45]. In this procedure, the graphical least absolute shrinkage and selection operator (gLASSO) is applied to estimate the structure of 100 regularized network models from sparse to dense and continues to fit an unregularized network for each of these models using gLASSO without regularization, but with zeroes constrained according to the network structure. Maximum likelihood estimation is used to obtain the unbiased estimates of the parameters. The Bayes information criterion (BIC) for each newly estimated model is computed iteratively, and the model with the lowest BIC is selected, thus enduring that the final model is attained when no edge can be removed or added to optimize the BIC. To visualize the network, the Fruchterman–Reingold algorithm was used. Here, the nodes with the highest centrality are drawn to the center of the network, and less important nodes are placed in the periphery [46], although the algorithm also functions to minimize the number of crossing edges.

A common centrality measure was obtained for each variable across the two estimated networks. Here, expected influence centrality [47] was calculated with standardized *z*-scores on the *x*-axis (low *z*-scores corresponds to low importance of the node in the network). Raw-score estimates are provided in the Supplementary Materials (see Figures S11–S13). Expected influence reflects greater importance of a node in the network. In addition, bridge centrality indices were obtained through the *networktools* package [24], displaying the most central nodes in bridging identified communities in the network. To investigate the community structure of the overall network, the spinglass algorithm [48] from the *igraph* package [49] was applied.

The accuracy of edge weights was assessed by nonparametric bootstrapping (1,000 iterations) 95% confidence intervals using the R package *bootnet* [50]. The stability of node strength was assessed using case-dropping subset *bootstrap* (1,000 iterations). In this procedure, the correlation between the original centrality indices and the centrality indices as obtained from smaller subsets, with up to 75% of participants dropped, is assessed. To quantify the stability of the indices, correlation stability coefficients (CS-coefficients) were calculated. A CS-coefficient indicates the maximum proportion of cases that can be dropped to retain, with 95% certainty, a correlation with the original centrality indices of 0.70 or higher. The CS-coefficient should preferably be 0.50 or higher [50].

## Results

Descriptive information about the nodes included in the network is presented in Supplementary Table S1 (prior to nonparanormal transformation). Two-thousand eight-hundred and sixty-eight parents were included in the study. The current sample included most female participants (79.5% female). All analyzed subgroups were, however, richly represented in the dataset given the number of participants (e.g., 587 males), and an adjusted poststratified and weighted sample has revealed identical results on the same group of participants in a previous study (see Johnson et al. [6]). Demographic information is provided in Table 1.

**Table 1.** Sample characteristics (*N* = 2,868).

Characteristic	*n* (%)
Parental role	
Mother	2,281 (79.5)
Father	587 (20.5)
Age	
21–30	359 (12.5)
31–44	1,728 (60.3)
45–64	768 (26.8)
65+	13 (0.5)
Marital status	
Married/cohabitating	2,447 (85.2)
Single parent	421 (14.7)
Number of children	
One child	2,407 (83.9)
Two or more children	461 (16.1)
Occupation	
Employed	2,241 (78.1)
Unemployed	627 (21.9)

### Network estimation

**Figure 1.**
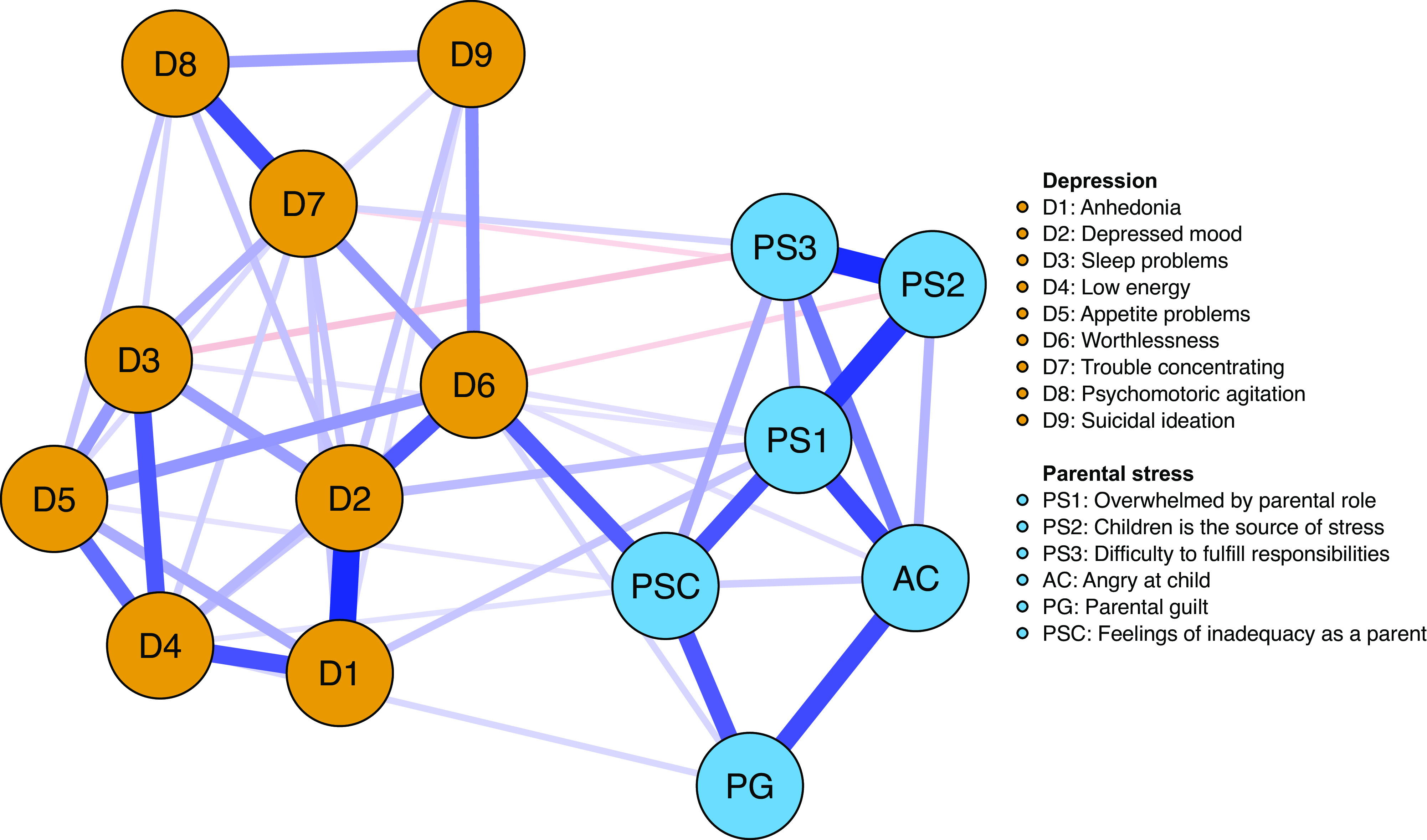
Network structure of depressive symptoms and parental stress. *Note:* Blue edges represent positive relations, whereas red edges represent negative relations.

**Figure 2.**
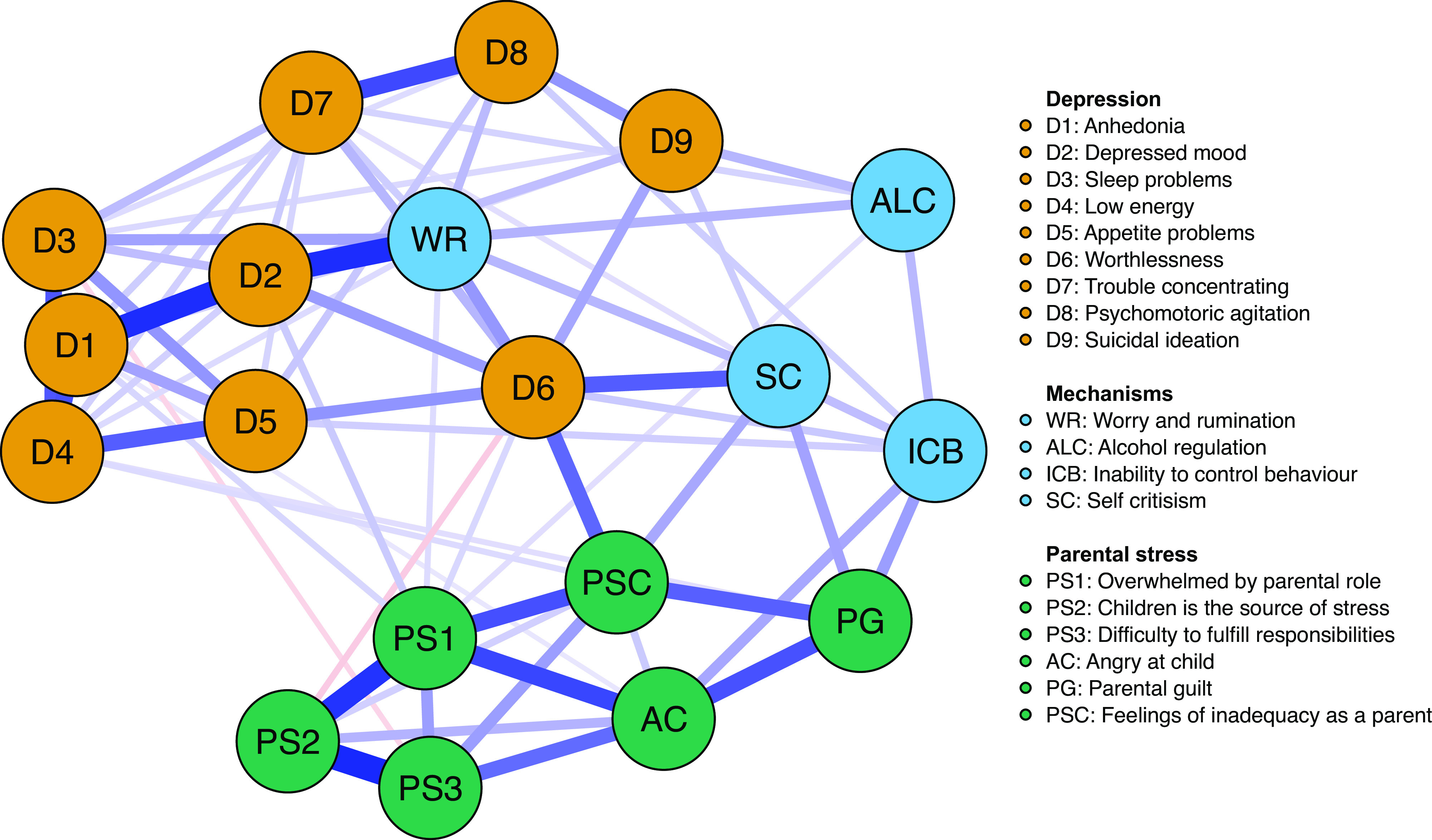
Network structure of depressive symptoms, parental stress, and mechanistic variables. *Note:* Blue edges represent positive relations, whereas red edges represent negative relations.

The resulting network structures, based on Spearman rank-order correlations, are visualized in Figures 1 and 2. The blue edges represent positive partial correlations between nodes, whereas the red edges represent negative partial correlations. In the first network (see Figure S4), particularly strong positive connections (see Figure S4) occur among Anhedonia (D1) and Depressed mood (D2); Overwhelmed by parental role (PS1) and identifying children as one’s main source to stress (PS2); in addition to PS2 and Difficulty to fulfill responsibilities (PS3). Notably, anger and frustration toward child(ren) (AC) was positively associated with all parental stress items, and additionally attached to feelings of worthlessness (D6). In bridging the two mental health states, the most remarkable positive connection appeared between D6 Worthlessness (D6) and Feelings of inadequacy as a parent (PSC).

**Figure 3.**
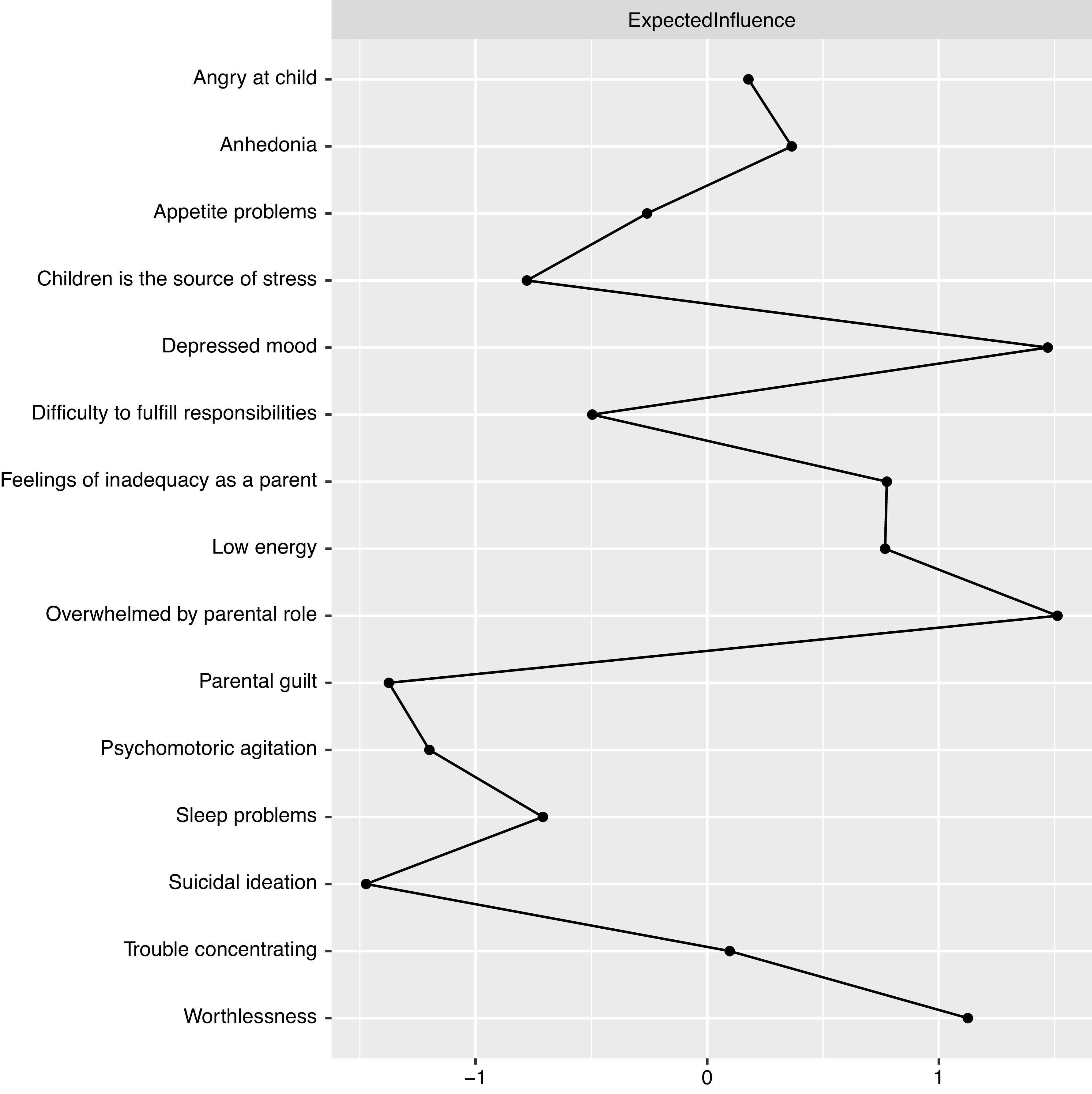
Expected influence estimates of the network including parental stress and depressive symptoms.

**Figure 4.**
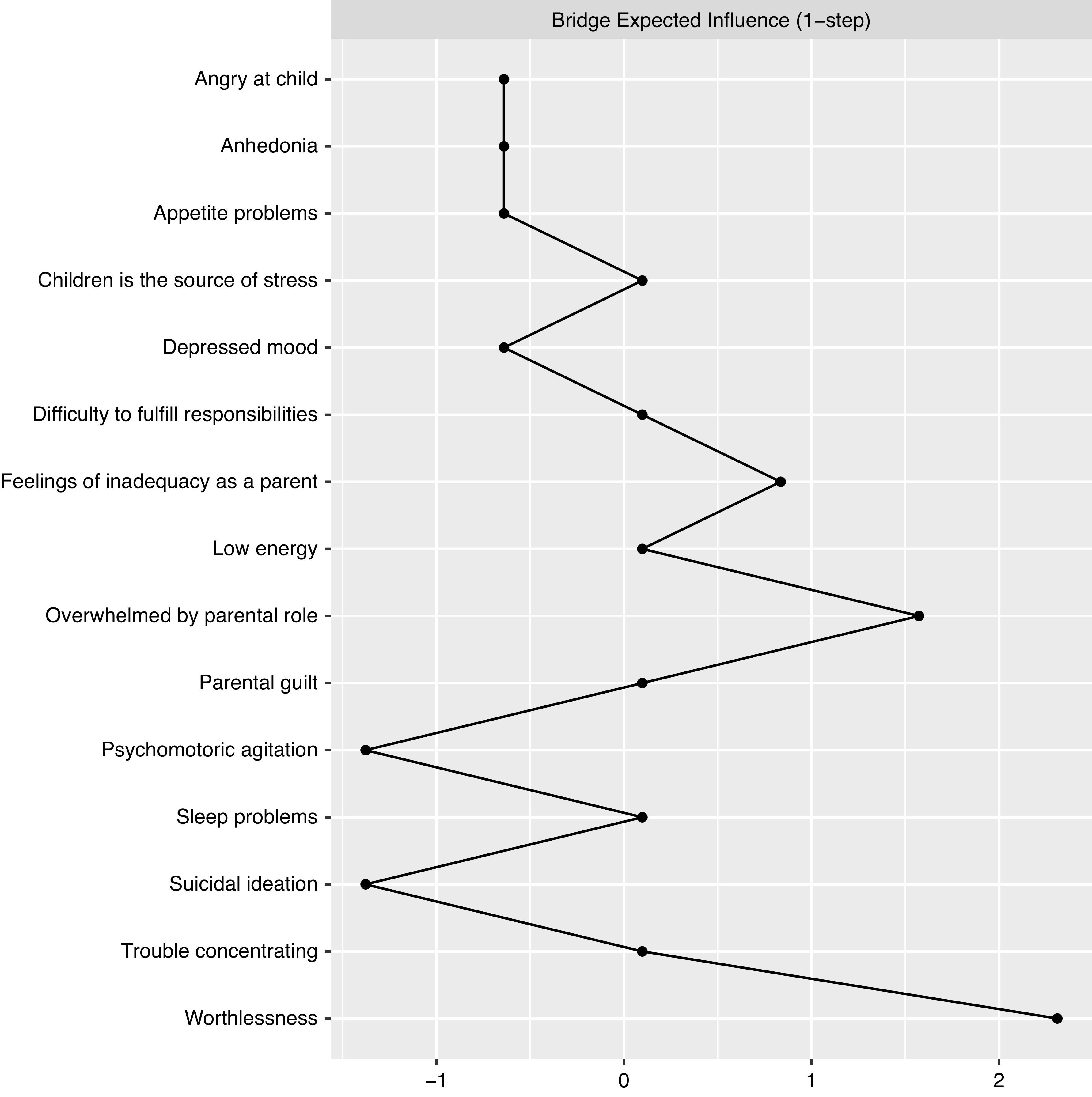
Bridge expected influence estimates of the network including parental stress and depressive symptoms.

**Figure 5.**
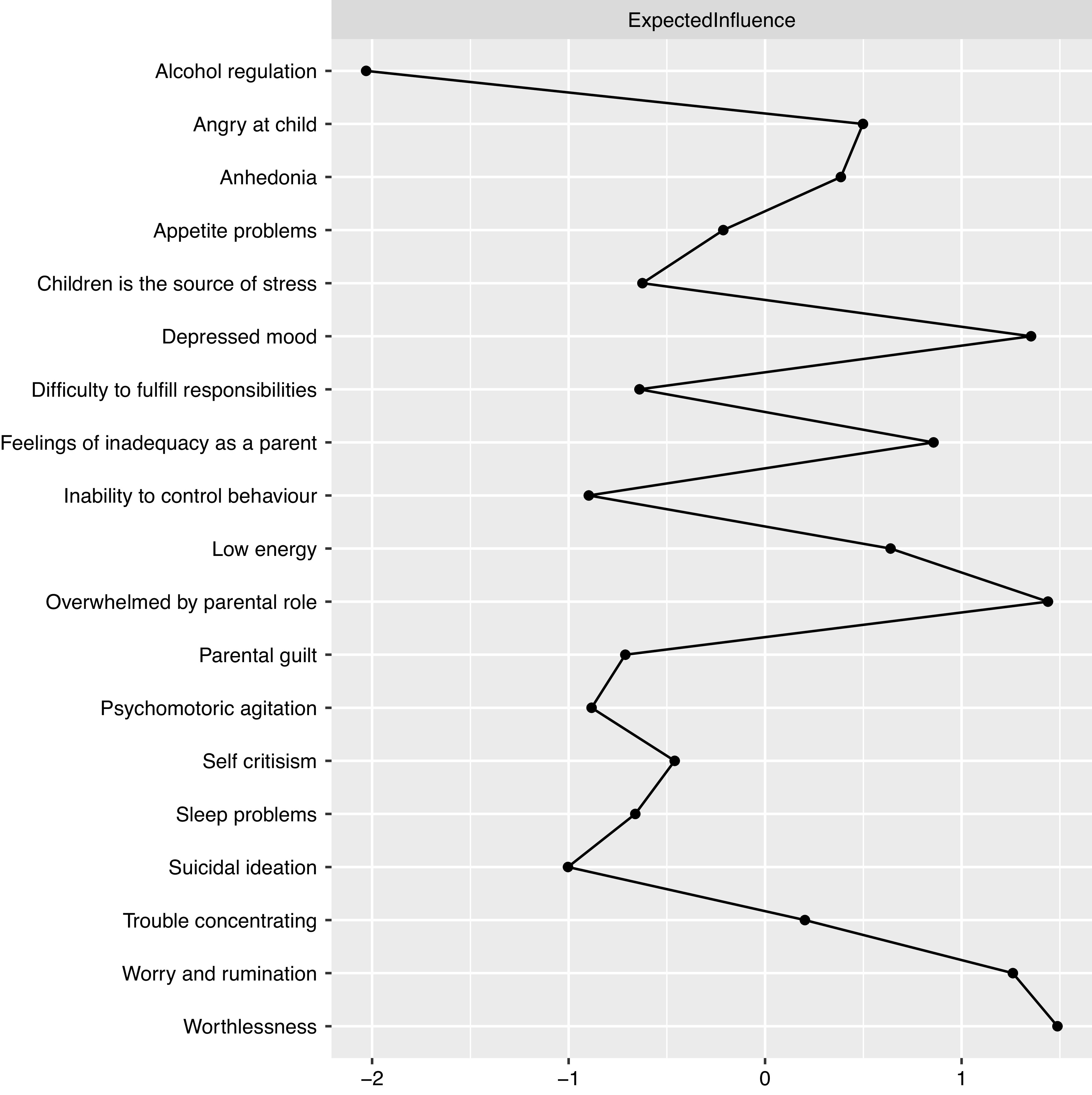
Expected influence estimates of the network including parental stress, depressive symptoms, and mechanistic variables.

The spin glass community analysis identified two clear-cut clusters (communities) in line with the visual representation of the network (Figure 1); one for parental stress items and one for the depressive symptoms. The expected influence and bridge expected influence for each node are presented in Figures 3–5. To access which node(s) are likely to be involved in bridging the distinct clusters of symptoms, bridge expected influence indexes (see Figure 4) were obtained for the first estimated network (Figure 1; symptoms only). Correspondingly, feeling worthless (D6) revealed the most substantial bridge expected influence index (see Figure 4), indicating the relevance of the node in bridging the two constructs together.

In both networks, the D6 node, which represents the depressive symptom feeling worthless, displays one of the highest expected influence (see Figures 3 and 5), revealing that the node has one of the highest sums of edge weights. This is reflected in the current networks (Figures 1 and 2) where Worthlessness (D6) has the most edges attached to it, including several nodes in the depressive cluster (within cluster) and parental stress cluster (between cluster). Other notable central nodes include Overwhelmed by the parental role (PS1) and Depressed mood (D2), both displaying high node expected influence (>1 *z*-score) in the parental stress and depressive network (see Figure 3). In the mechanistic network, in addition to the aforementioned nodes, WR displayed high expected influence (>1 *z*-score; see Figure 5).

For the second network including mechanistic variables (see Figure S5), the strongest positive links (edge weights) appeared among the aforementioned relationships (D1–D2, PS1–PS2, and PS3–PS2) as in the parental stress and depressive only network (Figure 1) and between WR and Anhedonia (D1). Among the other mechanistic variables, SC was most positively connected to worthlessness (D6). Using alcohol to cope with negative feelings (ALC) was most positively associated with suicidal ideation (D9), while inability to control behavior (ICB) was strongest and positively associated with AC and PG.

### Accuracy and stability

Figures and information regarding the accuracy and stability of the estimated networks and (bridge) expected influences are provided in the Supplementary Materials (see Supplementary Figures S1, S2, S6, and S7), and attested to the robustness of the network, indicating high stability of centrality indices and accurate edge weights. The CS-coefficients were revealed to be 0.75 for all expected influence and bridge expected influence estimates in the networks (as shown in Figures S6 and S7), hence implying that 75% of the data may be omitted to preserve with 95% certainty a correlation of 0.70 with the original dataset [51].

## Discussion

The current research provides findings regarding the specific links between parental stress components and depressive symptoms during the initial phase of the COVID-19 pandemic. The results reveal that co-occurrent parental stress and depressive symptoms display two separately clusters of symptoms, mainly connected through feelings of worthlessness. The strongest association with the parental stress community with worthlessness was the node measuring feeling inadequate as a parent. It may thus seem that the extra burden placed upon parents during the pandemic lockdown may have led to feelings of inadequacy, providing a maladaptive bridging opportunity with depressive symptomatology through worthlessness. This finding highlights the importance of everyday structures surrounding the parental population, which may not only alleviate them from stress, but possibly also function as a protection against feelings of inadequacy further associated with detrimental symptomatology.

Considering the relationships within the parental stress related items, feelings of being overwhelmed by the parental role (PS1) revealed the strongest strength centrality. Such observation is in line with the theoretical definition of parental stress; that parental stress arises when parents experience not being able to control or meet the demands that the parental role require [52]. Within the parents’ depressive cluster, depressed mood and worthlessness displayed highest expected influence. This is in line with recent studies regarding node centrality in depressive states in general populations (e.g., [53,54]), indicating that these symptoms may play similar roles in the parental population.

Although symptoms can create problematic states through their mutual interaction with one another, such as the co-occurring conditions between parental stress and depressive symptoms that were portrayed in Figure 1, symptoms often interact with mechanistic processes to emerge forth such states. The results from the mechanistic network (Figure 2) illustrate the substantial connection between excessive WR and depressive symptoms in parents during the pandemic, highlighting the key role of this mechanism. A central feature of WR involves the misguided notion that one is engaged in problem-solving through the process of rethinking one’s problems repeatedly. However, such repetitive cognitive processes have previously been associated with adverse mental health (e.g., [55]). Given the identified association between these processes with depressed mood and hopelessness (D2), it seems plausible that such circular activities may emerge into feeling stuck rather than function as true problem-solving activities. When access to several problem-focused coping strategies is limited (e.g., social support), people must handle the uncertainty of the situation and the associated pandemic stressors in a different manner. This could lead to excessive WR as a coping strategy aimed to reduce the stress which the individual experience. However, the present study reveals that such increases may amplify the pathways through which excessive WR are interconnected with detrimental symptomatology, putting individuals at greater risk during pandemic periods where phenomena such as worry increase substantially.

Additional mechanistic variable that displayed several associations with both parental stress and depressive symptoms was SC. While the node revealed relatively low overall expected influence, it relates to central nodes that were highlighted in the comorbid structure of parental stress and depression. As noted by Robinaugh et al. [47], researchers should not only consider the potential of changing one node based on its importance revealed solely by the expected influence estimates but include the possibility that interventions on low centrality nodes may promote consecutive interventions on high centrality nodes (e.g., changes in self-critical thinking may subsequently reduce feelings of worthlessness [highly central]). SC has previously been found to be particularly associated with parental stress and parental depression (e.g., [31]). During the initial phase of the pandemic, the demands of parents escalated rapidly, forcing mothers and fathers to stay at home with their children and provide necessary care and home schooling, while simultaneously conducting their own work from home. The increases in such demands could potentially lead parents to feel that they do not live up to or are able to respond with what is expected from them. Such elevated stress could furthermore lead to more self-critical thinking, here found to have distinct connections to several key symptoms of depression and parental stress (e.g., feelings of worthlessness and feeling inadequate in the parental role) that were found to link the adverse mental health states together. One initial randomized controlled trial has found that the use of cognitive reappraisal and self-compassion strategies in parents during the pandemic effectively reduces acute stress and parental mental health [56]. The use of such interventions seems appropriate for the present co-occurrence of the specific mental health complaints experienced by parents during the pandemic. Although the direction of associations remains unclear in cross-sectional networks, the outlined mechanism well-grounded in theory serve as starting points for future studies to investigate directionalities in studies using temporal design, a gap currently left to be filled in the parental pandemic literature.

Concerning the use of alcohol to regulate one’s feelings and cope with negative situations, this node was most strongly associated with suicidal ideation. Interpreting this against the backdrop in which concerns have been raised about suicidal ideation during the pandemic (e.g., [57,58]), forthcoming studies investigate this association more in depth. Finally, using alcohol to cope with emotional and situational difficulties revealed the lowest expected influence, indicating the least importance of this node in the overall network structure (less than −2 *z*-score). However, the network analysis of the mechanistic variables, depression, and parental stress unveils an interesting link between using alcohol to cope and the parental stress domain through impulse control difficulties in parents (i.e., ICB). This circuit should be investigated in greater detail to uncover if such temporal pathways do exist. In addition, this study is conducted on a sample of parents from the general population and not on a clinical population, subsequently leaving unanswered questions regarding the network structure of parents drawn from a clinical sample (e.g., depressed parents). It could be that using alcohol to cope would be a more prominent node in parents experiencing elevated depressive and parental stress symptoms. Future studies should investigate these symptoms and mechanisms in clinical samples.”

### Strengths and limitations

Several limitations should be considered when interpreting the results of the current study, including that not all participants were randomly selected, prestratification not conducted, and that some demographic subgroups were overrepresented (e.g., female participants). However, given our large sample, all subgroups were richly represented, and the sample was moreover revealed to be representative of the Norwegian population, in addition to revealing identical results through sensitivity analyses conducted solely on the randomly selected participants, in addition to adjusted and weighted sample with subgroups proportional to the population parameter [6]. Moreover, although the cross-sectional design has limitations regarding directionality and temporality [59], this article reveals associations between adverse symptoms and mechanistic processes which can effectively be manipulated with several therapeutic approaches (e.g., metacognitive therapy) [55]. This points toward the necessity of future intensive longitudinal studies to examine the within-person relations between nodes in the network, and how parental stress and depressive symptoms interact at the individual level over time. In addition, interventional studies are needed to examine whether manipulation of central nodes leads to change in the other nodes.

Nevertheless, several strengths should be noted. To the best of our knowledge, this is the first study examining the relationship between parental stress and depression using network analysis, and further incorporates actionable mechanisms subject to change. Instead of looking at the conditions as unitary construct, this research represent a more complex view of how depressive and parental stress symptoms are associated, specifically in a time where parental stress and depressive symptoms are heighted among parents in general. Furthermore, although these mental states are likely amplified during the pandemic, their pattern of interwovenness are likely similar in nonpandemic settings, making the presented results of utility in understanding the co-occurrence and bridging between parental stress, depression, and related mechanisms in general. A further strength of this study is that it included a large sample of parents experiencing identical social distancing protocols which were held constant during and prior to the measuring period. Finally, the large sample contributed to the accuracy, stability, and robustness of the network estimates.

### Future research

Considering doubt related to the validity and usefulness of centrality measures in psychological network analysis, and how these indices derived from cross-sectional studies map onto actual intervention targets [47,60], this finding should be further investigated in longitudinal studies involving dynamical systems approaches. Although aggregated cross-sectional approach provides important information regarding symptom interaction within a group (here 2,868 parents), such approaches lack the ability to detect the symptom structure and change in a single individual over time [61,62].

## Conclusion

As families continue to shelter at home, parents will continue to experience high symptom pressure. The cross-sectional findings display that the co-occurrence of parental stress and depression has specific pathways, was manifested through feelings of worthlessness, and has specific patterns of connection to important mechanisms of psychopathology, which should be examined further in future longitudinal studies.

## Data Availability

The received ethical approval granted by the Regional Committees for Medical and Health Research Ethics in Norway does not allow the data to be submitted in a public repository. In line with the ethics approval, the data are to be kept at a secure server accessible by the authors at the University of Oslo. Access to the data can be granted from the principal investigators Omid V. Ebrahimi (omideb@uio.no) and Sverre Urnes Johnson following ethical approval of a suggested project plan for the use of data from NSD and REK. A fully reproducible correlation matrix of the data used for this study is made available through the Open Science Framework: https://osf.io/62pfu/.

## Supplementary material

For supplementary material accompanying this paper visit http://dx.doi.org/10.1192/j.eurpsy.2021.2223. click here to view supplementary material
