# Supplementary material for: Feelings of worthlessness links depressive symptoms and parental stress: A network analysis during the COVID-19 pandemic
Source: Eur Psychiatry. 2021 Jul 27;64(1):e50. doi: 10.1192/j.eurpsy.2021.2223 (PMC8376856; doi:10.1192/j.eurpsy.2021.2223)
Supplement: Supplementary file 1 [file S0924933821022239sup001.docx]

**Supplementary Materials**

**Parental Stress Single Items**

*How much have the following statements applied to you during the last two weeks?*

“I feel overwhelmed by the responsibility of being a parent.” (PS1)

“The major source of stress in my life is my children.” (PS2)

“It is difficult to balance different responsibilities because of my children.” (PS3)

“I have been angrier and more frustrated then usual with my children”. (AC)

“I feel inadequate as a parent” (PSC)

“I feel guilty when I get angry with my children” (PG)

*The item was scored on a five-point Likert-Scale ranging from Strongly disagree (1) to Strongly agree (5).*

**Depressive symptoms**

*How often have you been bothered by the following over the past two weeks?*

“Little interest or pleasure in doing things?” (D1)

“Feeling down, depressed, or hopeless?” (D2)

“Trouble falling or staying asleep, or sleeping too much?” (D3)

“Feeling tired or having little energy?” (D4)

“Poor appetite or overeating?” (D5)

“Feeling bad about yourself — or that you are a failure or have let yourself or your family down?” (D6)

“Trouble concentrating on things, such as reading the newspaper or watching television?” (D7)

“Moving or speaking so slowly that other people could have noticed? Or so fidgety or restless that you have been moving a lot more than usual?” (D8)

“Thoughts that you would be better off dead, or thoughts of hurting yourself in some way?” (D9)

*The items were scored on a four-point Likert-Scale ranging from Not at all (0) to almost every day (3).*

**Mechanisms**

“I often criticize or blame myself if something is wrong” (SC)

*The item was scored on a four-point Likert-Scale ranging from Not at all (0) to almost every day (3).*

“How much time in the last week have you found yourself dwelling on or worrying about your problems?” (WR)

“How often in the last week have you used alcohol, pills or drugs to cope with your negative feelings or thoughts?” (ALC)

*The items were scored on a four-point Likert-Scale ranging from “nothing” (0)* to “*all the time” (8).*

“When I´m upset, I have difficulty controlling my behaviour” (ICB)

*The item was scored on a five-point Likert-Scale ranging from “almost never” (1)* to “*almost always” (5).*

| **Table S1.** Characteristics of all items. |  |  |  |  |  |
| --- | --- | --- | --- | --- | --- |
| Construct/node | Label | *M* | *SD* | Skewness | Kurtosis |
| **Depression** |  |  |  |  |  |
| Anhedonia | D1 | .82 | .82 | .96 | .63 |
| Depressed mood | D2 | .77 | .80 | 1.04 | .87 |
| Disturbed sleep | D3 | .88 | .94 | .90 | -.12 |
| Low energy | D4 | 1.25 | .87 | .59 | -.22 |
| Appetite problems | D5 | .86 | .96 | .89 | -.23 |
| Worthlessness | D6 | .81 | .91 | 1.03 | .28 |
| Trouble concentrating | D7 | .69 | .87 | 1.17 | .58 |
| Psychomotor agitation or impediment | D8 | .30 | .62 | 2.26 | 5.05 |
| Suicidal ideation | D9 | .12 | .42 | 4.09 | 19.28 |
| **Parental stress** |  |  |  |  |  |
| Overwhelmed by parental role | PS1 | 2.48 | 1.26 | .38 | -.1.01 |
| Child(ren) is the major source of stress | PS2 | 2.14 | 1.21 | .74 | -.62 |
| Difficulty to fulfil responsibilities | PS3 | 2.48 | 1.34 | .34 | -1.22 |
| Anger/frustration towards child(ren) | AC | 2.53 | 1.31 | .23 | -1.30 |
| Parental guilt | PG | 3.84 | 1.06 | -.83 | .19 |
| Feelings of inadequacy as a parent | PSC | 2.64 | 1.26 | .28 | -1.05 |
| **Mechanisms** |  |  |  |  |  |
| Worry and rumination | WR | 3.05 | 2.00 | .58 | -.51 |
| Alcohol regulation | ALC | .51 | 1.25 | 3.20 | 11.22 |
| Self-criticism | SC | 1.26 | .91 | .53 | -.45 |
| Inability to control behaviour | ICB | 1.58 | .80 | 1.69 | 3.35 |

**Figure Captions**

**Figure S1.** Pearson correlation plot of each nonparanormal-transformed variable.

*Note.* D1 = Anhedonia; D2 = Depressed mood; D3 = Disturbed sleep; D4 = Low energy; D5 = Appetite problems; D6 = Worthlessness; D7 = Trouble concentrating; D8 = Psychomotor agitation; D9 = Suicidal ideation; WR = Worry and rumination; ALC = Alcohol regulation; SC = Self-criticism; ICB = Inability to control behaviour; PS1 = Overwhelmed by parental role; PS2 = Child(ren) is the major source of stress; PS3 = Difficulty to fulfil responsibilities; AC = Anger/frustration towards child(ren); PG = Parental guilt; PI Feelings of inadequacy as a parent.

**Figure S2.** Stability of edge weights in the parental stress and depression network.

**Figure S3.** Stability of edge weights in the network including parental stress, depressive symptoms, and mechanisms.

**Figure S4.** Edge weights difference test in the network of parental stress and depressive symptoms.

**Figure S5.** Edge weights difference test in the network including parental stress, depressive, and mechanistic variables.

**Figure S6.** Stability of expected influence and bridge expected in the parental stress and depressive symptom network.

**Figure S7.** Stability of expected influence and bridge expected influence in the parental stress depressive symptoms, and mechanisms network.

**Figure S8.** Centrality difference test of expected influence in the network with depressive symptoms and parental stress.

**Figure S9.** Centrality difference test of bridge expected influence in the network with depressive symptoms and parental stress.

**Figure S10.** Centrality difference test of expected influence in the network with depressive symptoms, parental stress, and mechanistic variables.

**R-code**

#

#Load packages

library("glmnet")

library("bootnet")

library("qgraph")

library("dplyr")

library("psychonetrics")

library("NetworkComparisonTest")

library("huge")

library("corrplot")

library("matrixcalc")

library("networktools")

library("igraph")

#Set working directory

setwd("~/Documents/UiO /Nettverk og psykose/Artikkel_Nettverk_Parental stress og dep./Final /R")

# Import csv file

Data1 <- read.csv("COVID-19 - Parents - Wide - OPPDATERT.csv")

View(Data1)

#NETWORK 1: Parenting stress and depressive symptoms

#Select variabels

Data3 <- Data1 %>% dplyr::select(phq1_t1, phq2_t1, phq3_t1, phq4_t1, phq4_t1, phq5_t1, phq6_t1, phq7_t1, phq8_t1, phq9_t1, dps_pstress1_t1, dps_pstress2_t1, dps_pstress3_t1, angryatchild_t1, parentalguiltang_t1, parentalselfcrit_t1)

#Nonparanormal transformation

data_npt<-as.data.frame(huge.npn(Data3))

summary(data_npt)

#Rename variables

Data4 <- data_npt %>%

rename(

D1 = phq1_t1,

D2 = phq2_t1,

D3 = phq3_t1,

D4 = phq4_t1,

D5 = phq5_t1,

D6 = phq6_t1,

D7 = phq7_t1,

D8 = phq8_t1,

D9 = phq9_t1,

PS1 = dps_pstress1_t1,

PS2 = dps_pstress2_t1,

PS3 = dps_pstress3_t1,

AC = angryatchild_t1,

PG = parentalguiltang_t1,

PSC = parentalselfcrit_t1,

)

# Node names:

namesObject <- c("Anhedonia", "Depressed mood", "Sleep problems", "Low energy",

"Appetite problems","Worthlessness", "Trouble concentrating",

"Psychomotoric agitation", "Suicidal ideation",

"Overwhelmed by parental role", "Children is the source of stress",

"Difficulty to fulfill responsibilities","Angry at child",

"Parental guilt",

"Feelings of inadequacy as a parent")

# Grouping object for visualization

groupObject <- c(rep("Depression", 9), rep("Parental stress", 6))

# Correlations between variables

corM <- cor(Data4)

pdf("3) Corrplot.pdf")

#Plotting correlations

col <- colorRampPalette(c("#BB4444", "#EE9988", "#FFFFFF", "#77AADD", "#4477AA"))

corrplot(corM, method = "color", col = col(200),

type = "upper", order = "alphabet", number.cex = .7,

addCoef.col = "black",

tl.col = "black", tl.srt = 90, diag = FALSE)

dev.off()

#check if matrix is positive definite

corpcor::is.positive.definite(cor_auto(Data4)) #TRUE

## Use goldbricker function to search for potential "bad pairs"

gb <- goldbricker(Data4, p = 0.05, method = "hittner2003", threshold=0.25, corMin=.50)

gb #Suggested reductions: Less than 25 % of correlations are significantly different for the following pairs:

# [1] "No suggested reductions" ### ALL OK ####

#Thanks to Blanchard at al.(2021) from which this part of the code (line 69 - 90) is inspired by and adapted from with permission

# ESTIMATE NETWORK: PARENTAL STRESS & DEPRESSION

netParents <- estimateNetwork(

Data4,

default = "ggmModSelect",

corMethod = "spearman")

summary(netParents)

# Plot network and save it as PDF:

graph1 <- plot (netParents,

layout = "spring",

theme = "colorblind",

groups = groupObject,

nodeNames = namesObject,

legend.cex = 0.35,

width = 6,

height = 5,

filetype = "pdf", filename = "1) Nettverk _ Stress and Depression")

#COMMUNITY DETECTION

#transform graph1 to igraph

g <- as.igraph(graph1, attributes=TRUE)

sgc <- spinglass.community(g)

sgc$csize

sgc$membership # community membership

## 2 2 2 2 2 2 2 2 2 1 1 1 1 1 1 # clearly seperated in two communities (Stress & depression)

# Make adjacency matrix for bridge centrality

net_matrix <- as_adjacency_matrix(

g,

type = c("both", "upper", "lower"),

attr = NULL,

edges = FALSE,

names = TRUE,

sparse = igraph_opt("sparsematrices")

)

### Plot - bridge expected influence and expected influence

b <- bridge(net_matrix, communities=groupObject, directed=FALSE, nodes = namesObject)

b

#Make pdf file for Bridge centrality

pdf("4) Bridge Expected Influence.pdf")

#Plotting Bridge centrality

plot(b, include=c("Bridge Expected Influence (1-step)"), theme_bw=FALSE, zscore = TRUE, order = "alphabetical")

dev.off()

#Plotting pdf file for Raw Scores Bridge Centrality

pdf("4) Bridge Expected Influence_RAWSCORES.pdf")

#Plotting Bridge centrality Raw Scores

plot(b, include=c("Bridge Expected Influence (1-step)"), theme_bw=FALSE, zscore = FALSE, order = "alphabetical")

dev.off()

#Make pdf file for centrality

#Centrality plot

pdf("5) Centrality plot.pdf")

centralityPlot(netParents, theme_bw=FALSE,labels = namesObject, include = c("expectedInfluence"), decreasing = TRUE, scale = "z-scores")

dev.off()

#Centrality plot - RAWSCORES

pdf("5) Centrality plot_RAWSCORES.pdf")

centralityPlot(netParents, theme_bw=FALSE,labels = namesObject, include = c("ExpectedInfluence"), decreasing = TRUE, scale = "raw")

dev.off()

#BOOTNET AND STABILITY

bootEW_nonparametric <- bootnet(

netParents,

nBoots = 1000,

nCores = 1,

statistics = "all",

communities = groupObject,

split0 = TRUE

)

pdf("6)bootEW_nonparametric.pdf")

plot(bootEW_nonparametric,

labels = FALSE,

order = "sample",

plot = "interval")

dev.off()

# PDF object for edge differences

pdf("7) Indirect - Edge Weight Differences - ggmModSelect.pdf")

# Plot edge differences

plot(bootEW_nonparametric,

plot = "difference",

onlyNonZero = TRUE,

order = "sample")

# Close PDF device:

dev.off()

# Significance tests:

pdf("8) ExpectedInfluence - significance test - ggmModSelect.pdf")

# Compare significant differences in expected influence

plot(bootEW_nonparametric, statistics="ExpectedInfluence", plot="difference")

# Close PDF device:

dev.off()

pdf("8) BrigdeExpectedInfluence - significance test - ggmModSelect.pdf")

# Compare significant differences in bridge expected influence

plot(bootEW_nonparametric, statistics = "bridgeExpectedInfluence", plot="difference")

# Close PDF device:

dev.off()

#STABILITY

boot_casedrop <- bootnet(

netParents,

nBoots = 1000,

nCores = 1,

type = "case",

statistics = c("expectedInfluence", "bridgeExpectedInfluence"),communities=groupObject)

pdf("9) Stability_casedrop.pdf")

corStability(boot_casedrop)

plot(boot_casedrop, c("expectedInfluence", "bridgeExpectedInfluence"), communities=groupObject)

dev.off()

#NETWORK 2: Parenting stress and depressive symptoms and mechanisms

#Select variabels

Data_mec <- Data1 %>% dplyr::select(phq1_t1, phq2_t1, phq3_t1, phq4_t1, phq4_t1, phq5_t1, phq6_t1, phq7_t1, phq8_t1, phq9_t1, dps_pstress1_t1, dps_pstress2_t1, dps_pstress3_t1, angryatchild_t1, parentalguiltang_t1, parentalselfcrit_t1, cas_1_t1, cas_5_t1, ders_ic_t1, selfcritical_t1)

#Nonparanormal transformation

data_npt_mec<-as.data.frame(huge.npn(Data_mec))

summary(data_npt_mec)

#Rename variables

Data_mec_net <- data_npt_mec %>%

rename(

D1 = phq1_t1,

D2 = phq2_t1,

D3 = phq3_t1,

D4 = phq4_t1,

D5 = phq5_t1,

D6 = phq6_t1,

D7 = phq7_t1,

D8 = phq8_t1,

D9 = phq9_t1,

PS1 = dps_pstress1_t1,

PS2 = dps_pstress2_t1,

PS3 = dps_pstress3_t1,

AC = angryatchild_t1,

PG = parentalguiltang_t1,

PSC = parentalselfcrit_t1,

WR = cas_1_t1,

ALC = cas_5_t1,

ICB = ders_ic_t1,

SC = selfcritical_t1

)

# Node names:

namesObject_mec <- c("Anhedonia", "Depressed mood", "Sleep problems", "Low energy",

"Appetite problems","Worthlessness", "Trouble concentrating",

"Psychomotoric agitation", "Suicidal ideation",

"Overwhelmed by parental role", "Children is the source of stress",

"Difficulty to fulfill responsibilities","Angry at child", "Parental guilt",

"Feelings of inadequacy as a parent",

"Worry and rumination","Alcohol regulation",

"Inability to control behaviour", "Self critisism"

)

# Grouping object for visualization

groupObject_mec <- c(rep("Depression", 9), rep("Parental stress", 6), rep("Mechanisms", 4))

# Correlations between variables

corM_mec <- cor(Data_mec_net)

pdf("10) Corrplot_mec.pdf")

#Plotting correlations

col <- colorRampPalette(c("#BB4444", "#EE9988", "#FFFFFF", "#77AADD", "#4477AA"))

corrplot(corM_mec, method = "color", col = col(200),

type = "upper", order = "alphabet", number.cex = .7,

addCoef.col = "black",

tl.col = "black", tl.srt = 90, diag = FALSE)

dev.off()

#check if matrix is positive definite

corpcor::is.positive.definite(cor_auto(Data_mec_net)) #TRUE

## Use goldbricker function to search for potential "bad pairs"

gb <- goldbricker(Data_mec_net, p = 0.05, method = "hittner2003", threshold=0.25, corMin=.50)

gb #Suggested reductions: Less than 25 % of correlations are significantly different for the following pairs:

# [1] "No suggested reductions" ### ALL OK ####

#ESTIMATE NETWORK: PARENTING STRESS & DEPRESSION & MECHANISMS

netParentsMec <- estimateNetwork(

Data_mec_net,

default = "ggmModSelect",

corMethod = "spearman")

# Plot network and save it as PDF:

graph2 <- plot (netParentsMec,

layout = "spring",

theme = "colorblind",

groups = groupObject_mec,

nodeNames = namesObject_mec,

legend.cex = 0.35,

width = 6,

height = 5,

filetype = "pdf", filename = "2) Nettverk _ Stress and Depression and Mechanism")

# Make adjacency matrix for bridge centrality

net_matrix_mec <- as_adjacency_matrix(

g2,

type = c("both", "upper", "lower"),

attr = NULL,

edges = FALSE,

names = TRUE,

sparse = igraph_opt("sparsematrices")

)

b2 <- bridge(net_matrix_mec, communities=groupObject_mec, directed=FALSE, nodes = namesObject_mec)

# Plot - bridge centrality and centrality

pdf("11) Bridge Expected Influence_Mec.pdf")

#Plotting Bridge centrality

plot(b2, include=c("Bridge Expected Influence (1-step)"), theme_bw=FALSE, zscore = TRUE, order = "alphabetical")

dev.off()

#Centrality plots

pdf("12) Centrality Plots_Mec.pdf")

centralityPlot(netParentsMec, theme_bw=FALSE,labels = namesObject_mec, include = c("ExpectedInfluence"), decreasing = TRUE, scale = "z-scores")

dev.off()

#Centrality plots - RAW SCORES

pdf("12) Centrality Plots_Mec_RAWSCORES.pdf")

centralityPlot(netParentsMec, theme_bw=FALSE,labels = namesObject_mec, include = c("ExpectedInfluence"), decreasing = TRUE, scale = "raw")

dev.off()

#BOOTNET AND STABILITY

bootEW_nonparametric_mec <- bootnet(

netParentsMec,

nBoots = 1000,

nCores = 1,

statistics = "all",

communities = groupObject_mec,

split0 = TRUE

)

pdf("13) bootEW_nonparametric_mec.pdf")

plot(bootEW_nonparametric_mec,

labels = FALSE,

order = "sample",

plot = "interval")

dev.off()

# PDF object for edge differences

pdf("14) Indirect - Edge Weight Differences - ggmModSelect_mec.pdf")

# Plot edge differences

plot(bootEW_nonparametric_mec,

plot = "difference",

onlyNonZero = TRUE,

order = "sample")

# Close PDF device:

dev.off()

# Finding node differences

# PDF object for expected influence differences

pdf("15) ExpectedInfluence - significance test - ggmModSelect.pdf")

# Compare significant differences in expected influence

plot(bootEW_nonparametric_mec, statistics="ExpectedInfluence", plot="difference")

# Close PDF device:

dev.off()

#STABILITY

boot_casedrop_mec <- bootnet(

netParentsMec,

nBoots = 1000,

nCores = 1,

type = "case",

statistics = c("ExpectedInfluence", "bridgeExpectedInfluence"),communities=groupObject_mec)

pdf("16) Stability_casedrop.pdf")

corStability(boot_casedrop_mec)

plot(boot_casedrop_mec, c("ExpectedInfluence", "bridgeExpectedInfluence"), communities=groupObject_mec)

dev.off()
